# Postoperative transverse sternal nonunion with a chest wall defect managed by a tibial locking plate and a Gore-Tex dual mesh membrane: a case report

**DOI:** 10.1186/s13019-021-01730-5

**Published:** 2021-12-06

**Authors:** Tomaz Malovrh, Tomaz Stupnik, Boris Podobnik, Jurij Matija Kalisnik

**Affiliations:** 1 Department of Traumatology, University Medical Centre Ljubljana, Zaloska cesta 7, 1000 Ljubljana, Slovenia; 2 Medical Faculty, University of Ljubljana, Ljubljana, Slovenia; 3 Department of Thoracic Surgery, University Medical Centre Ljubljana, Ljubljana, Slovenia; 4 Cardiac Surgery, Cardiovascular Center, Klinikum Nürnberg - Paracelsus Medical University, Nuremberg, Germany

**Keywords:** Sternal nonunion, Plate fixation, Transverse thoracosternotomy, Clamshell incision, Chest wall reconstruction

## Abstract

**Background:**

Transverse sternal nonunion is a rare but disabling complication of chest trauma or a transverse sternotomy. Fixation methods, mainly used to manage the more common longitudinal sternal nonunion, often fail, leaving the surgical treatment of transverse nonunion to be a challenge.

**Case presentation:**

We present a case of a highly-disabling, postoperative chest wall defect resulting from transverse sternal nonunion after a transverse thoracosternotomy (clamshell incision) and a concomitant rib resection. Following unsuccessful surgical attempts, the sternal nonunion was fixed with a tibial locking plate and bone grafted, while the post-rib resection chest defect was reconstructed with a Gore-Tex dual mesh membrane. Adequate chest stability was achieved, enabling complete healing of the sternal nonunion and the patient’s complete recovery.

**Conclusion:**

We believe it is important to address both in the rare case of combined postoperative transverse sternal nonunion and the chest wall defect after rib resection. A good outcome was achieved in our patient by fixing the nonunion with an appropriately sized and shaped locking plate with bone grafting and covering the chest defect with a dual mesh membrane.

## Background

Sternal nonunion is a rare, potentially disabling complication, which occurs after a sternotomy or a sternal fracture [1]. The vast majority of sternal nonunions are longitudinal and result from healing disturbances after a median sternotomy. Transverse sternal nonunion is far less common, occurring predominantly after a sternal fracture or after a transverse sternotomy, a procedure which is rarely performed [1, 2]. The condition is defined by the lack of radiographic signs showing osseous healing of the sternum, accompanied by localized pain and tenderness for at least 3 months [3]. Persistent fracture line—with or without callus formation or even bone resorption and a gap across the sternum—is usually visible on lateral X-ray radiograph. Computed Tomography (CT) scan can be used to confirm the absence of osseous union between the fragments, especially when the radiographs are inconclusive [4]. Surgical treatment should be considered when the patient with a sternal nonunion is symptomatic [5–7]. However, it is important to note that surgical methods developed and successfully used to manage the more common longitudinal sternal nonunion are generally not applicable to the transverse nonunion [1, 2].

## Case presentation

**Fig. 1:**
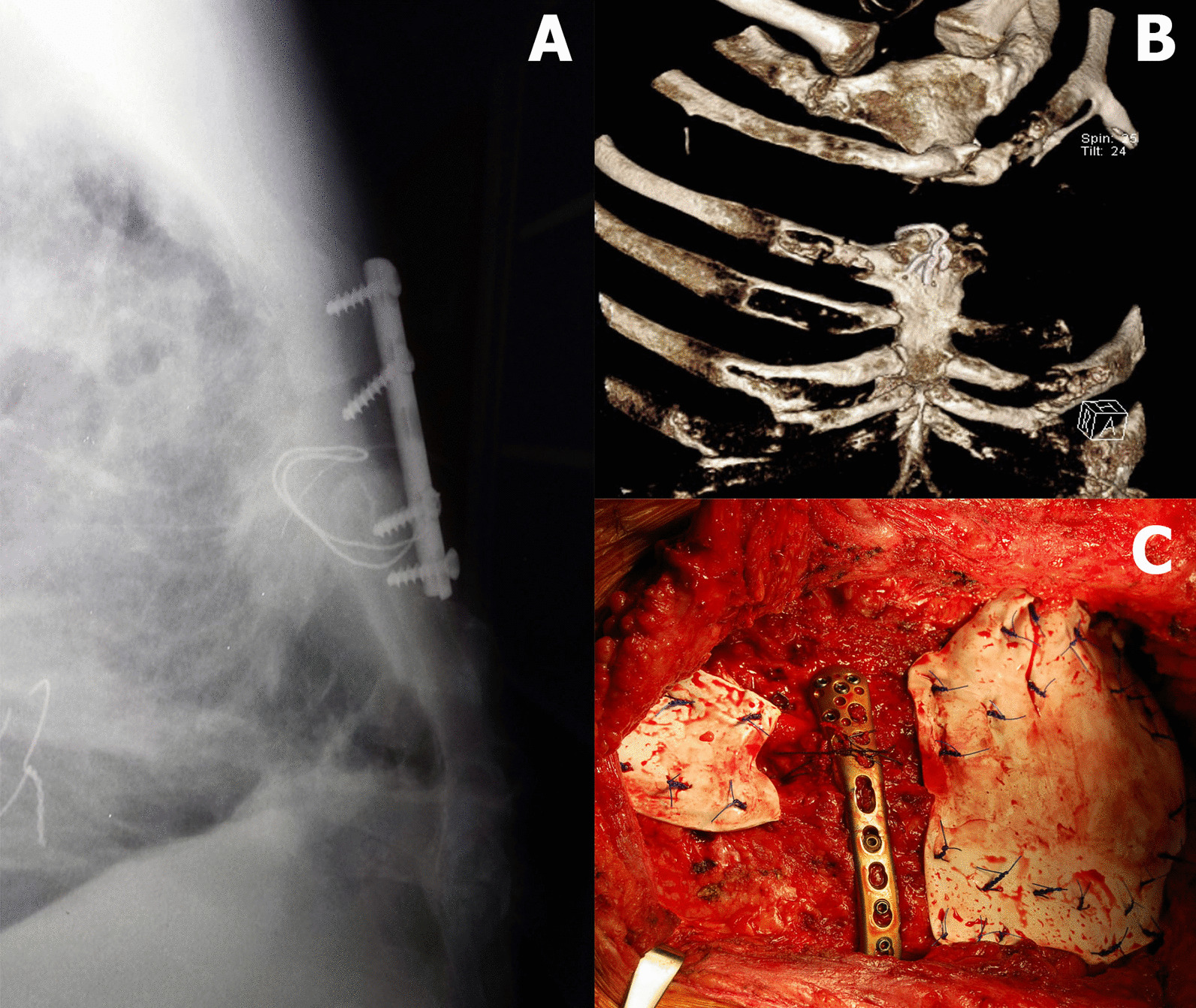
**a** A failed fixation of the transverse sternal nonunion with one-third tubular plate and tension wiring. **b** 3D reconstruction of the CT showing a big chest wall defect resulting from sternal nonunion and rib resection. **c** Intraoperative view of the sternal fixation with a locking tibial plate and closure of the remaining chest wall defects with two Gore-Tex dual mesh membranes

A 50-year-old man was primarily operated on for extensive type B aortic dissection with acute bowel ischemia. The portion of the descending aorta from the origin of the left subclavian artery to just below the superior mesenteric artery was replaced by a tubular graft through an extended posterolateral thoracotomy. The patient recovered well; however, 10 years later, he was diagnosed with an aortic arch aneurysm and severe dilatation of the proximal descending aorta at the site of the anastomosis with descending tubular graft. Transverse thoracosternotomy (clamshell incision) was used to approach and replace the ascending aorta, the aortic arch, and the proximal part of the previous tubular graft in a single session as a re-do procedure. To facilitate the performance of the distal graft to graft anastomosis, the third and part of the fourth ribs on the left side were resected in addition to the transverse sternotomy. The sternotomy was closed with tension wires. Dehiscence of the transverse sternotomy developed during the early postoperative period. Two attempts of tension re-wiring followed by fixation with one-third tubular plate and conventional screws were performed to address the dehiscence, which all failed in a few weeks (Fig. 1a).

Three years after the last unsuccessful revision surgery, the patient returned to our clinic symptomatic with pain and an unstable chest, with his lung herniating through the chest wall defect. Radiographs and a CT scan revealed an extensive chest deformation resulting from a widely displaced sternal nonunion, a bone defect of the sternum resulting from multiple operations, and the resections of the third and fourth ribs on the left side of the chest (Fig. 1b).

The patient was operated again by a team of cardiothoracic and orthopaedic trauma surgeons. An extensive adhesiolysis, the removal of the remaining wires, and debridement of the scar tissue were done. The sternal fragments were refreshed until the healthy bone and approximated, while the remaining 1 cm-large defect was filled with a structural iliac crest bone autograft. The fragments were then compressed by bone reduction clamps. The fixation was done using a titanium locking compression plate designed for the distal medial tibia (DepuySynthes, West Chester, Pennsylvania). The plate was intraoperatively shaped to match the markedly changed anatomy of the proximal sternum and fixed using 3.5 mm locking screws. The wider shape of the plate with multiple screw holes provided a good purchase in a smaller portion of the deformed proximal fragment. All the screws were carefully inserted under "finger control" as not to protrude at the back of the sternum. In the meantime, a 2 mm-thick, 20 × 10 cm Gore-Tex dual mesh membrane was attached to the ribs to cover the remaining defect caused by rib resection on the left side. The membrane was attached by using interrupted trans-costal sutures from heavy polypropylene. More than 60 sutures were placed along the two adjacent ribs above and below the defect to achieve the required strength. Another membrane of a 10 × 5 cm size was applied to the remaining soft tissue defect in the second intercostal space on the right side (Fig. 1c). The wound was then closed, the thoracic cavity was drained, and two drains were placed subcutaneously to prevent haematoma or a subsequent seroma formation.

**Fig. 2:**
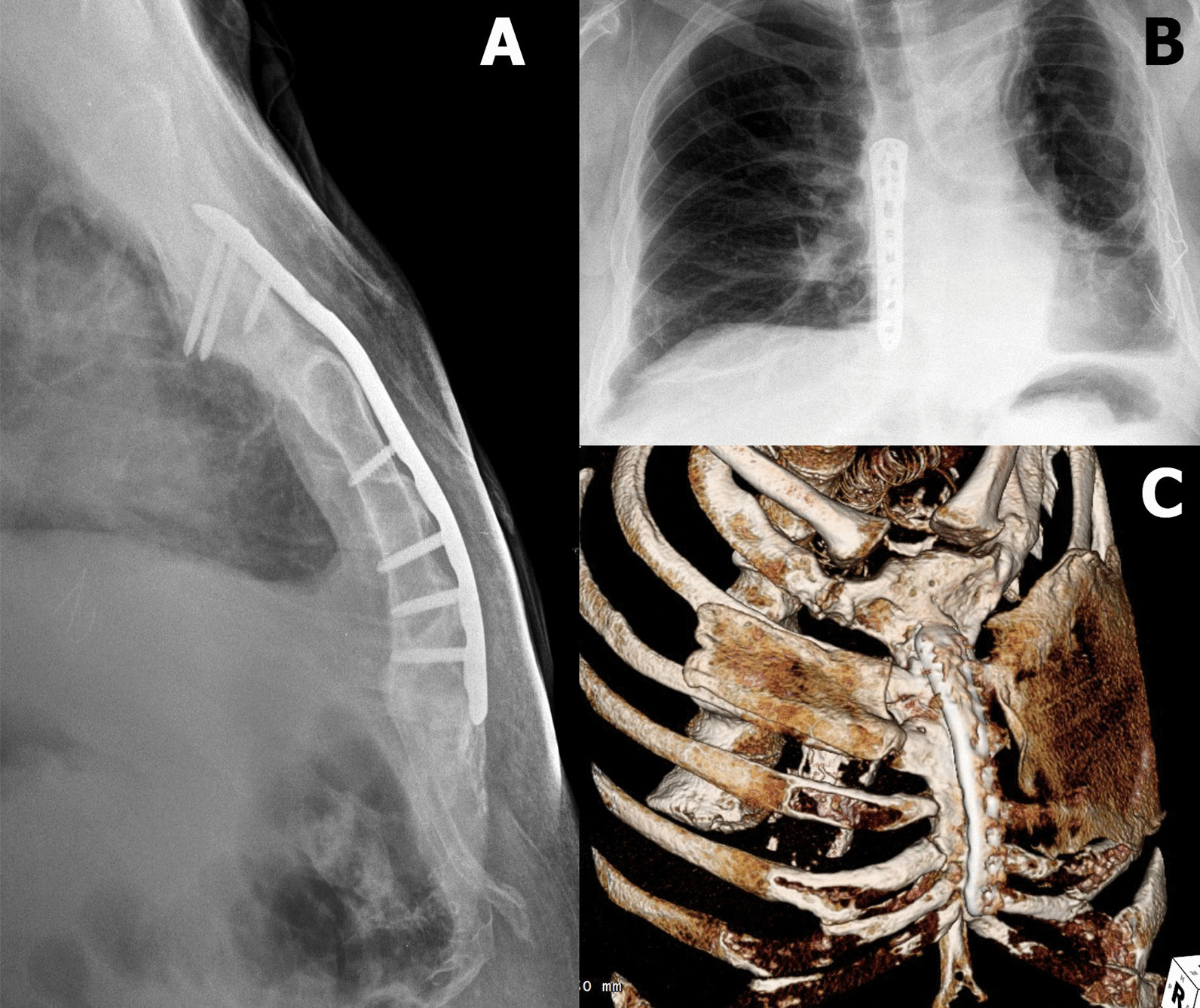
Chest X-ray. **a** lateral; **b** AP view. **c** 3D reconstruction of the CT 1 year postoperatively showing healing of the sternum and good Gore-Tex dual mesh membranes position

The postoperative recovery of the patient was uneventful. The chest X-ray and the CT scan, performed 1 year after the operation showed the complete healing of the sternum and good positions of the membranes (Fig. 2). After 3 years, the chest remained stable with no pain or lung herniation. There were also no implant-related problems and, therefore, no need for plate removal.

## Discussion and conclusions

Transverse sternal nonunion is a rare but disabling complication that occurrs mainly after a traumatic sternal fracture or a rarely-performed transverse sternotomy [1]. The rate of nonunion is less than 1% after sternal fractures, while the condition becomes more common after a transverse sternotomy, with the rate of 6.8%, and thus represents an important chronic complication [7, 8]. A transverse thoracosternotomy (known as a Clamshell incision) is typically used in double-lung transplantations as it provides excellent access to the heart, lungs, and the great vessels [8, 9]. Due to multidirectional movements with tension and compression forces that act in all planes, fixation of the transverse sternotomy, sternal fracture, or transverse nonunion remains a challenge [1, 2]. There is limited published data on transverse sternal nonunion, especially after a sternotomy, mostly involving case reports or small case series [1, 10, 11]. Different fixation materials and techniques were described to fix transverse fractures, osteotomies, or nonunions, with tension wiring and plating being the most commonly performed [2, 7, 10]. Tension wires, which are still typically used to close a median sternotomy, often fail when used for a transverse sternotomy closure, which also was the case in our patient during the early postoperative period. A sternal notching technique should be considered when a transverse sternotomy is performed. The technique was described as the modification of sternal transection from transverse to inverted V-shaped. It allows for a more precise fit of the sternal fragments at closure, provides better stability, and possibly lowers the transverse sternal nonunion rate [12]. When using cerclage wires, the crossed wiring closure technique reduced the incidence of sternal dehiscence compared to the uncrossed technique [13]. In their comparative study, Qin-Yun Ma and co-workers showed a significantly higher sternal healing rate in the plated group compared to the tension-wired group after a transverse sternotomy [14]. Biomechanical advantages of sternal plating were previously shown in a cadaveric study [15]. Other authors also reported good functional outcomes of sternal plating with a high rate of union, mainly after using one or two parallel locking plates [2, 6, 8, 9, 11, 16, 17]. In contrast to conventional plating, locking implants with screws locked in the plate (angularly stable fixation) better resist continuous multidirectional forces that act on the construct during breathing. Additionally, monocortical locking screws can be a safer option to prevent an iatrogenic injury to the vital retrosternal structures. But on the other hand, they provide less purchase than bicortical locking screws, especially in a weaker bone [6, 11]. A review of the published literature done by Schulz et al. [18] showed good results for the most locking plate systems that were used and a review from Klei et al. from 2018 confirmed that plating was the most common type of fixation after sternal fractures (83%), with a reasonable consolidation and low complication rate [10, 18]. In our case, the patient had a symptomatic postoperative chest wall defect because of transverse sternal nonunion combined with a rib resection. The nonunion was previously unsuccessfully treated with tension wiring and one-third tubular plate fixed with conventional, non-locking screws. Following available published data, we fixed the nonunion with a locking plate. Due to the anatomical conditions, we did not use one or two regular longitudinal 3.5 mm locking plates. Instead, we decided to use a single, stronger locking plate that is typically used to fix distal tibial fractures, which we shaped to the bone. As our patient was tall and obese, the plate appeared to be well sized. The broad ending of the plate with multiple locking screws applied in different directions provided a strong purchase in a deformed proximal fragment. The sternal defect was also bone grafted with a tricortical iliac crest autograft. Bone grafting was advocated for and performed in some other published cases of transverse sternal nonunion with a bone defect [1, 2, 19, 20]. Common late complications of the sternal plating are local pain and irritation caused by the prominent material, requiring removal of the plate in 15.4% and 27% of the patients, respectively [5, 11]. There were no implant-related problems in our case and therefore no need for plate removal.

A Gore-Tex dual mesh membrane was additionally used in our patient to cover the chest wall defect after rib resection. Gore-Tex dual mesh membranes were shown to be a good option for reconstructing chest wall defects, especially after wide surgical resections [21–23]. By using the membranes, we successfully closed the remaining defects and treated the lung herniation. Furthermore, we believe that membranes also diminished the load applied on the plate during continuous chest movements and possibly contributed to the prevention of an early plate failure.

We reviewed the literature for similar cases and could only find a few small series and case reports describing the successful combination of titanium plates with Gore-Tex dual mesh membranes to achieve a stable chest that allows the complete range of respiratory movements, most of them being used after wide surgical resection of the thorax [22, 24]. However, we could not find any reports of similar treatment in such an extensive postoperatively deformed chest, resulting from widely displaced sternal nonunion and rib resection. To our knowledge, this is also the first reported case of a specific tibial plate successfully being used to fix a transverse sternal nonunion.

In conclusion, we presented a rare case of a patient with a symptomatic postoperative chest wall defect resulting from transverse sternal nonunion after a transverse thoracosternotomy (clamshell incision) and concomitant rib resection. We believe that addressing both the transverse sternal nonunion and the chest wall defect after rib resection with a properly sized and shaped locking plate with bone grafting and a dual mesh membrane, respectively, was important for a favourable outcome in our patient.

## Data Availability

The authors declare that the data supporting the findings of this study are available within the article.

## Abbreviation

CT: Computed tomography

## References

[1] Bertin KC, Rice RS, Doty DB, Jones KW (2002). Repair of transverse sternal nonunions using metal plates and autogenous bone graft. Ann Thorac Surg.

[2] Queitsch C, Kienast B, Voigt C, Gille J, Jürgens C, Arndt PS (2011). Treatment of posttraumatic sternal non-union with a locked sternum-osteosynthesis plate (TiFix). Injury.

[3] Mayba II (1985). Non-union of fractures of the sternum. J Bone Jt Surg Ser A.

[4] Coons DA, Pitcher JD, Braxton M, Bickley BT (2002). Sternal nonunion. Orthopedics.

[5] Harston A, Roberts C (2011). Fixation of sternal fractures: a systematic review. J Trauma Inj Infect Crit Care.

[6] Saka N, Watanabe Y, Sasaki G, Iida M, Kawano H (2018). Nonunion of the sternum treated with cervical locking plate: a case report. J Orthop Sci.

[7] Wu LC, Renucci JD, Song DH (2005). Sternal nonunion: a review of current treatments and a new method of rigid fixation. Ann Plast Surg.

[8] Motomura T, Bruckner B, La Francesca S, Mittelhaus S, Chike-Obi C, Leon-Becerril J (2011). Experience of sternal secondary closure by means of a titanium fixation system after transverse thoracosternotomy. Artif Organs.

[9] Gandy KL, Moulton MJ (2008). Sternal plating to prevent malunion of transverse sternotomy in lung transplantation. Ann Thorac Surg.

[10] Klei DS, de Jong MB, Öner FC, Leenen LPH, van Wessem KJP (2019). Current treatment and outcomes of traumatic sternal fractures—a systematic review. Int Orthop.

[11] Kalberer N, Frima H, Michelitsch C, Kloka J, Sommer C (2020). Osteosynthesis of sternal fractures with double locking compression plate fixation: a retrospective cohort study. Eur J Orthop Surg Traumatol.

[12] Lonchyna VA (1999). Sternal “notching” improves clamshell incision. Oper Tech Thorac Cardiovasc Surg.

[13] Koster TD, Ramjankhan FZ, Van De Graaf EA, Luijk B, Van Kessel DA, Meijer RCA (2013). Crossed wiring closure technique for bilateral transverse thoracosternotomy is associated with less sternal dehiscence after bilateral sequential lung transplantation. J Thorac Cardiovasc Surg.

[14] Ma QY, Zhu YJ, Pang LW, Chen G, Chen J, Chen ZM (2011). Application of the titanium plate fixation system in sternum transverse incisions. Am Surg.

[15] Ozaki W, Buchman SR, Iannettoni MD, Frankenburg EP (1998). Biomechanical study of sternal closure using rigid fixation techniques in human cadavers. Ann Thorac Surg.

[16] Gloyer MA, Frei HC, Hotz TK, Käch KP (2011). Osteosynthesis of traumatic manubriosternal dislocations and sternal fractures with a 3.5/.40 mm fixed-angle plate (LCP). Arch Orthop Trauma Surg.

[17] Zhao Y, Yang Y, Gao Z, Wu W, He W, Zhao T (2017). Treatment of traumatic sternal fractures with titanium plate internal fixation: a retrospective study. J Cardiothorac Surg.

[18] Schulz-Drost S, Oppel P, Grupp S, Schmitt S, Carbon RT, Mauerer A (2015). Surgical fixation of sternal fractures: preoperative planning and a safe surgical technique using locked titanium plates and depth limited drilling. J Vis Exp.

[19] Hoschtitzky JA, Khalid R, Au J, Sampath S (2005). Open reduction and internal fixation of a non-united transverse sternal fracture. Inj Extra.

[20] Severson EP, Thompson CA, Resig SG, Swiontkowski MF (2009). Transverse sternal nonunion, repair and revision: a case report and review of the literature. J Trauma Inj Infect Crit Care.

[21] Akiba T, Marushima H, Nogi H, Kamiya N, Kinoshita S, Takeyama H (2012). Chest wall reconstruction using Gore-Tex® dual mesh. Ann Thorac Cardiovasc Surg.

[22] Hamad AM, Marulli G, Bulf R, Rea F (2009). Titanium plates support for chest wall reconstruction with Gore-Tex® dual mesh after sternochondral resection. Eur J Cardio Thorac Surg.

[23] Nagayasu T, Yamasaki N, Tagawa T, Tsuchiya T, Miyazaki T, Nanashima A (2010). Long-term results of chest wall reconstruction with DualMesh. Interact Cardiovasc Thorac Surg.

[24] Berthet JP, Wihlm JM, Canaud L, Joyeux F, Cosma C, Hireche K (2012). The combination of polytetrafluoroethylene mesh and titanium rib implants: an innovative process for reconstructing large full thickness chest wall defects. Eur J Cardio-Thorac Surg.

